# Draft Genome Sequences of *Mycobacterium kansasii* Clinical Strains

**DOI:** 10.1128/genomeA.00406-17

**Published:** 2017-06-01

**Authors:** Paulina Borówka, Jakub Lach, Zofia Bakuła, Jakko van Ingen, Aleksandra Safianowska, Anna Brzostek, Jarosław Dziadek, Dominik Strapagiel, Tomasz Jagielski

**Affiliations:** a Department of Anthropology, University of Łódź, Łódź, Poland; b University of Łódź, Łódź, Poland; c Department of Applied Microbiology, Faculty of Biology, Institute of Microbiology, University of Warsaw, Warsaw, Poland; d Department of Medical Microbiology, Radboud University Medical Center, Nijmegen, The Netherlands; e Department of Internal Medicine, Pulmonology, and Allergology, Warsaw Medical University, Warsaw, Poland; f Mycobacterium Genetics and Physiology Unit, Institute of Medical Biology, Polish Academy of Sciences, Lódź, Poland; g Biobank Lab, Department of Molecular Biophysics, University of Lódź, Lódź, Poland

## Abstract

*Mycobacterium kansasii* is a nontuberculous mycobacterial (NTM) pathogen, frequently isolated from clinical samples and responsible for a large part of NTM infections in the human population. Here, we report the draft genome sequences of 12 *M. kansasii* strains isolated from clinical and host-associated sources from the Netherlands, Germany, and Poland.

## GENOME ANNOUNCEMENT

*Mycobacterium kansasii* is one of the most significant nontuberculous mycobacterial (NTM) pathogens recovered from clinical specimens and is responsible for a large part of NTM infections in the human population (1, 2). However, culturing of *M. kansasii* from human tissues may not necessarily represent true infection but rather a nonpathogenic colonization. Grouping of *M. kansasii* into seven genetically similar subtypes (I to VII) adds more complexity to the clinical and epidemiological relevance of *M. kansasii* isolations. Of the *M. kansasii* subtypes, type I has been isolated most frequently from human sources and involved in the causation of NTM disease (3–5).

To better explore the genetic diversity of *M. kansasii* and recognize the effect of genetic background on the bacterial phenotype, the first large-scale *M. kansasii* genome sequencing project has been launched (6). The project operates on a diverse pool of *M. kansasii* strains, of both environmental and clinical origin, from different geographical locales. The key purpose of the research is to identify molecular markers, specifically those associated with the pathogenic phenotype. This could lead to a new method for a fast and reliable diagnosis of *M. kansasii* disease.

Here, we announce the draft genome sequences of 12 clinical and host-associated *M. kansasii* strains, representing three subtypes—namely, I, II, and VI. The strains were recovered from pulmonary clinic patients in the Netherlands, Germany, and Poland and were sequenced, as summarized in Table 1.

**TABLE 1:** Genome features and GenBank accession numbers of selected strains

Strain	Subtype	Source^a^	Host disease	BioSample no.	Accession no.	Genome size (bp)	Coverage (×)
NLA001000927	I	Sputum	NTM disease	SAMN06339040	CP019883	6,421,275	35
NLA001001166	VI	Sputum	None	SAMN06215600	MWKW00000000	6,443,486	33
NLA001001128	II	BAL	None	SAMN06339041	CP019888	6,251,123	46
NLA001000449	I	Sputum	NTM disease	SAMN06339042	MWKX00000000	6,440,784	39
NLA001000521	I	BAL	NTM disease	SAMN06339043	CP019884	6,64,9816	44.6
ATCC 25221	I	Sputum	NTM disease	SAMN06339044	CP019886	6,462,452	36
B11073207	II	Sputum	None	SAMN06339045	CP019887	6,123,476	34.6
B11063838	II	BAL	None	SAMN06339046	MWKY00000000	6,126,434	44
7728	I	BW	NTM disease	SAMN06339047	MWQA00000000	6,463,923	33
6200	I	Sputum	NTM disease	SAMN06339048	CP019885	6,421,364	39
7744	I	BW	NTM disease	SAMN06339049	MWKZ00000000	6,434,062	45.8
2193	II	BW	None	SAMN06368474	MWKV00000000	6,254,980	33

a Abbreviations: BAL, bronchoalveolar lavage; BW, bronchial washing.

Genomic DNA was extracted and purified using the protocol of van Embden et al. (7). Genotyping was performed by means of PCR-restriction endonuclease analysis for the *hsp65* and *tuf* genes, as previously described (4, 8). Illumina paired-end libraries were prepared with the Nextera XT kit, in accordance with the manufacturer’s instructions. Whole-genome shotgun sequencing was performed on the MiSeq platform (Illumina) at a read length of 2 × 250 bp.

A total of 1,261,159, 1,274,864, 1,348,130, 1,609,452, 1,549,556, 1,498,034, 1,616,934, 1,386,880, 1,714,844, 1,390,906, 1,207,915, and 1,151,718 reads were obtained for strains NLA001000927, NLA001001166, NLA001001128, NLA001000449, NLA001000521, ATCC 25221, B11073207, B11063838, 7728, 6200, 7744, and 2193, respectively. The draft genomes of genotype I strains were *de novo* assembled with SPAdes software version 3.7.1 (9), with manual editing using FA_TOOL (10). The resulting assemblies were scaffolded with MeDuSa software (11). *De novo* assemblies of genotype II and VI strains were prepared using SPAdes version 3.7.1, Abyss version 2.0.2 (12), and Ray version 2.3.1 (13) simultaneously. To improve assembly contiguity, the obtained sets of contigs were integrated using CISA version 1.3 (14). The scaffolding process was performed by CONTIGuator (15), while gap closing was done with GapBlaster version 1.1.1 (16). Annotation was carried out automatically using the NCBI Prokaryotic Genome Annotation Pipeline (http://www.ncbi.nlm.nih.gov/genome/annotation_prok). The total lengths of the contigs for each genome, along with some basic clinical data regarding the strains, are presented in Table 1. 

### Accession number(s).

The accession numbers of the draft genome sequences of the *M. kansasii* strains reported here are provided in Table 1.
